# Fc**γ**RIIB is a T cell checkpoint in antitumor immunity

**DOI:** 10.1172/jci.insight.135623

**Published:** 2021-02-22

**Authors:** Clara R. Farley, Anna B. Morris, Marvi Tariq, Kelsey B. Bennion, Sayalee Potdar, Ragini Kudchadkar, Michael C. Lowe, Mandy L. Ford

**Affiliations:** 1 Department of Surgery and; 2 Department of Hematology and Oncology, Emory University School of Medicine, Atlanta, Georgia, USA.; 3 Winship Cancer Institute, Emory University, Atlanta, Georgia, USA.

**Keywords:** Immunology, Oncology, Adaptive immunity, T cells

## Abstract

In the setting of cancer, T cells upregulate coinhibitory molecules that attenuate TCR signaling and lead to the loss of proliferative capacity and effector function. Checkpoint inhibitors currently in clinical use have dramatically improved mortality from melanoma yet are not effective in all patients, suggesting that additional pathways may contribute to suppression of tumor-specific CD8^+^ T cell responses in melanoma. Here, we show that FcγRIIB, an inhibitory Fc receptor previously thought to be exclusively expressed on B cells and innate immune cells, is upregulated on tumor-infiltrating effector CD8^+^ T cells in an experimental melanoma model and expressed on CD8^+^ T cells in patients with melanoma. Genetic deficiency of *Fcgr2b* resulted in enhanced tumor-infiltrating CD8^+^ T cell responses and significantly reduced tumor burden. Adoptive transfer experiments of *Fcgr2b*^–/–^ tumor antigen-specific T cells into FcγRIIB-sufficient hosts resulted in an increased frequency of tumor-infiltrating CD8^+^ T cells with greater effector function. Finally, FcγRIIB was expressed on CD8^+^ memory T cells isolated from patients with melanoma. These data illuminate a cell-intrinsic role for the FcγRIIB checkpoint in suppressing tumor-infiltrating CD8^+^ T cells.

## Introduction

Metastatic melanoma is an aggressive form of skin cancer, with an estimated 91,270 new cases and 9320 disease-related deaths per year (1). Over the last 30 years, the incidence of melanoma has continued to rise steadily. Although patients with advanced disease (stages III and IV) have traditionally had a poor prognosis (1), recent advances in the utilization of immune-based therapies have revolutionized the management of metastatic melanoma (2). It is now well appreciated that cancer results in an increase in T cell expression of coinhibitory receptors, ultimately attenuating these cells’ proliferative capacity and effector function (3). Recent research has shown that reinvigoration of tumor-specific CD8^+^ T cell responses through immune modulation is an effective strategy to reduce tumor burden and decrease mortality. In particular, treatment of patients with cancer with mAbs targeting T cell coinhibitory pathways, such as CTLA-4 and PD-1 checkpoint inhibitors, has resulted in enhanced immune function against tumor cells and prolonged survival in patients with metastatic melanoma (4). However, not all patients are responsive to blockade of these specific checkpoint inhibitors, raising the possibility that additional novel checkpoint inhibitors exist and could be therapeutically targeted to further improve survival in patients with melanoma (5).

Fc receptors are a heterogeneous group of transmembrane immunoglobulin family molecules responsible for mediating a wide range of processes including antibody-dependent cellular cytotoxicity, endocytosis of antigen-antibody immune complexes, cytokine and chemokine production, and B cell development and homeostasis (6). Much like T cell costimulatory and coinhibitory molecules, the balance of activating and inhibitory Fc receptor signaling determines the outcome of antibody engagement of these receptors. Although there are several activating Fc receptors, FcγRIIB is the only known inhibitory Fc receptor and is conserved in both mice and humans (7). It is expressed by many immune cell types including B cells, dendritic cells, macrophages, and granulocytes (8). The effect of FcγRIIB inhibitory signaling on B cells has been extensively studied and reagents designed to target this inhibitory pathway are making their way into clinical trials for autoimmunity (6, 7, 9, 10). Significant work has also been done to elucidate the role of FcγRIIB on B cells and other antigen-presenting cells in the setting of cancer. For example, FcγRIIB on malignant B cells has been shown to accelerate internalization of targeting mAbs (e.g., the anti-CD20 mAb rituximab), hampering their ability to induce antibody-dependent cellular cytotoxicity and phagocytosis, and reducing their therapeutic efficacy (11). Use of an antagonistic FcγRIIB antibody 6G11 has been shown to prevent this internalization and preserve the therapeutic potential of rituximab (12). In contrast, FcγRIIB on B cells may potentiate the immunostimulatory effects of certain mAbs, specifically antibodies targeting CD40, OX40, and GITR, in an ITIM-independent manner by facilitating mAb cross-linking (13–16). Studies using a mouse B16 melanoma model have further demonstrated a higher efficacy of the protective IgG2a antibody TA99 in *Fcgr2b*^–/–^ mice compared with WT mice (17), as demonstrated by an approximately 30-fold greater reduction in the volume of lung metastases in *Fcgr2b*^–/–^ mice compared with WT mice.

However, exceedingly little is known about the role of FcγRIIB in directly modulating T cell expansion, survival, or effector function during antitumor immunity. Although early immunology literature speculated that T cells may express Fc receptors (18–20), for the past few decades the consensus has been that T cells do not express these molecules (6). However, several RNA-Seq data sets have suggested that FcγRIIB is expressed within CD8^+^ T cell populations (21, 22); and although a 2014 study demonstrated protein expression by flow cytometry of FcγRIIB on CD8^+^ T cells in response to microbial and viral infections (23), the functional significance of FcγRIIB on CD8^+^ T cells was unclear. We recently demonstrated a cell-autonomous role for FcγRIIB in regulating graft-specific CD8^+^ T cell responses in the context of transplantation, and during the course of those studies, we discovered that adoptive transfer of *Fcgr2b^–/–^* OT-I T cells into recipients of B16-OVA melanoma tumors resulted in an approximately 40% reduction in tumor volume by day 14 (24). However, the role of FcγRIIB-expressing CD8^+^ T cells in modulating antigen-specific CD8^+^ T cell accumulation and effector function within tumors is unknown. Here, we demonstrate that FcγRIIB is upregulated on a subset of activated, tumor-infiltrating CD8^+^ T cells and plays a cell-autonomous role in the suppression of tumor-infiltrating CD8^+^ T cells in a mouse model of melanoma. Our findings illuminate the role of a potentially novel checkpoint inhibitor in potently regulating CD8^+^ antitumor immunity in the setting of melanoma.

## Results

### FcγRIIB was expressed by memory CD8^+^ T cells in a murine cancer model.

**Figure 1:**
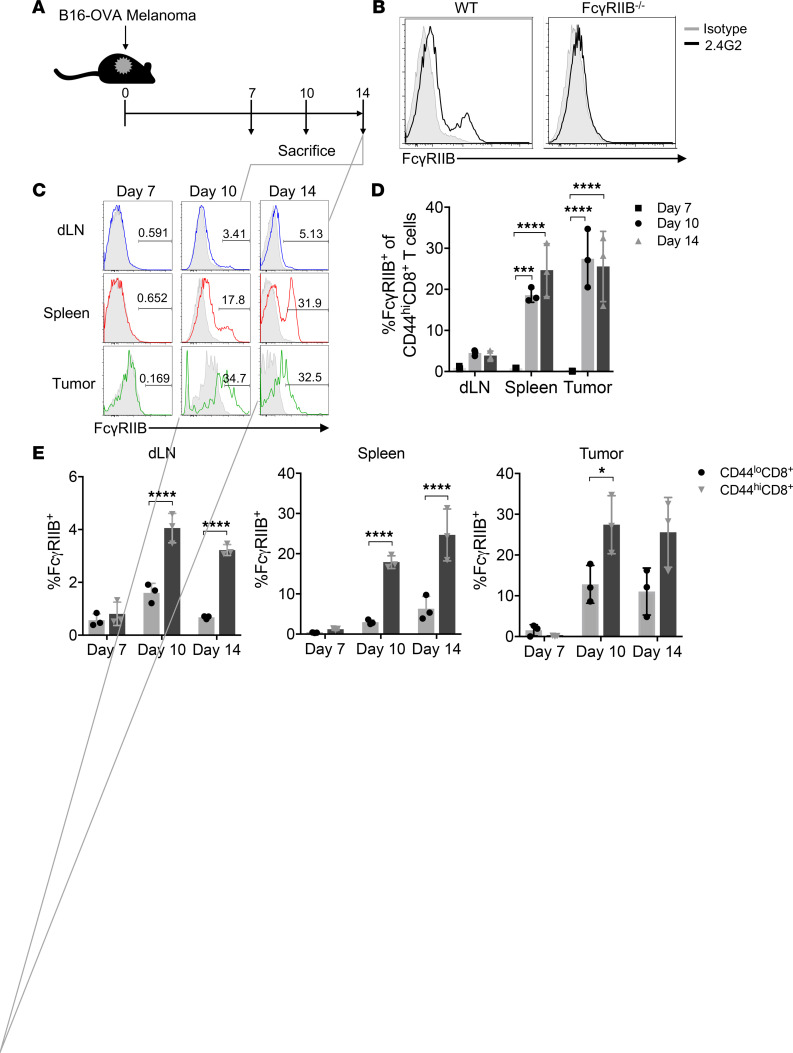
FcγRIIB is expressed on memory CD8^+^ T cells in a murine cancer model. (**A**) Schematic of experimental design: 10^6^ B16-OVA melanoma cells were injected into the subcutaneous tissue of the right flank of C57BL/6 mice on day 0. Spleen, draining lymph node (dLN), and tumor were harvested on days 7, 10, and 14. (**B**) CD44^hi^CD8^+^ T cells from the spleen of C57BL/6 (WT) and *Fcgr2b*^–/–^ mice were stained with anti-FcγRIIB/III (clone 2.4G2) (black) and isotype control (gray) on day 10 after tumor inoculation. (**C**) Representative histograms of FcγRIIB expression (clone 2.4G2) on CD44^hi^CD8^+^ T cells in the dLN (blue), spleen (red), and tumor (green) on days 7, 10, and 14 after tumor inoculation. Corresponding isotype controls are shown in gray. (**D**) Summary data of FcγRIIB expression (clone 2.4G2) on CD44^hi^CD8^+^ T cells in the dLN (blue), spleen (red), and tumor (green) on days 7, 10, and 14 after tumor inoculation. (**E**) Frequencies of FcγRIIB expression on CD44^lo^CD8^+^ (black) and CD44^hi^CD8^+^ (gray) in the dLN, spleen, and tumor. Data shown are representative of three independent experiments; *n* = 3–5 mice/group/experiment. Two-way ANOVA with multiple comparisons, *P < 0.05, ***P < 0.0005, ****P < 0.0001.

To determine whether FcγRIIB is expressed on CD8^+^ T cells during the immune response to melanoma, we first inoculated WT C57BL/6 (B6) mice with a B16-OVA melanoma cell line. Draining lymph node (dLN), spleen, and tumor were harvested on days 7, 10, and 14, respectively (Figure 1A). FcγRIIB surface expression was measured using the 2.4G2 mAb. Because 2.4G2 is known to bind both FcγRIIB and FcγRIII, we first confirmed that the staining we observed on CD8^+^ T cells was in fact specific to FcγRIIB by staining cells from both WT and *Fcgr2b*^–/–^ mice and compared the results with an isotype control. Our data demonstrated a lack of 2.4G2 staining on *Fcgr2b*^–/–^ CD8^+^ T cells (Figure 1B), confirming that the staining we observed on WT CD8^+^ T cells was FcγRIIB and not FcγRIII expression. Interestingly, FcγRIIB was not expressed on WT CD8^+^ T cells in the dLN, spleen, or tumor on day 7; however, FcγRIIB expression was identified on a subset of CD44^hi^CD8^+^ T cells in the dLN, spleen, and tumor on days 10 and 14 (Figure 1, C and D). Importantly, FcγRIIB was expressed on a significantly greater frequency of CD44^hi^CD8^+^ cells compared with CD44^lo^CD8^+^ cells (Figure 1E), indicating that FcγRIIB was expressed predominantly by activated CD8^+^ T cells. FcγRIIB was also expressed on a greater frequency of CD44^hi^CD8^+^ T cells in the tumor and spleen compared with the draining LN (Figure 1E).

### FcγRIIB was associated with 2B4 and PD-1 expression on effector CD8^+^ T cells.

**Figure 2:**
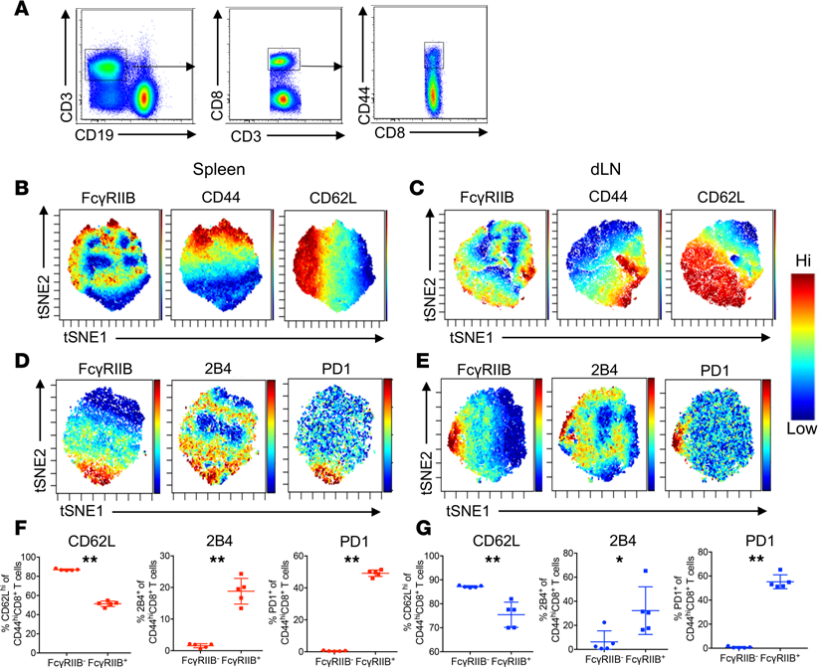
FcγRIIB is associated with 2B4 and PD-1 expression on CD44^hi^CD8^+^ T cells in the spleen and dLN in mice with melanoma. 10^6^ B16-OVA melanoma cells were subcutaneously injected into the right flank of C57BL/6 mice on day 0. Spleen and dLN were harvested on day 14. (**A**) Using conventional fluorescence-based flow cytometry, bulk CD3^+^CD8^+^ T cells (**B** and **C**) and CD44^hi^CD8^+^ CD3^+^ T cells (**D** and **E**) were gated and exported as FCS files for viSNE analysis. (**B**) viSNE maps showing the intensity of FcγRIIB, CD44, and CD62L expression on CD8^+^ T cells in the spleen. (**C**) viSNE maps showing intensity of FcγRIIB, CD44, and CD62L expression on CD8^+^ T cells in the dLN. (**D**) viSNE maps showing intensity of expression of FcγRIIB, 2B4, and PD-1 on CD44^hi^CD8^+^ T cells in the spleen. (**E**) viSNE maps showing intensity of expression of FcγRIIB, 2B4, and PD-1 on CD44^hi^CD8^+^ T cells in the dLN. (**F**) Summary data of the frequency of CD62L^hi^, 2B4^+^, and PD1^+^FcγRIIB^+^ and FcγRIIB^–^ CD44^hi^CD8^+^ T cells in the spleen. (**G**) Summary data of the frequency of CD62L^hi^, 2B4^+^, and PD1^+^FcγRIIB^+^ and FcγRIIB^–^ CD44^hi^CD8^+^ T cells in the dLN. Data shown are representative of 2 independent experiments; *n* = 5 mice/group/experiment. Mann-Whitney *U* test, *P < 0.05, **P < 0.005. dLN, draining lymph node.

To further explore the expression of FcγRIIB on CD8^+^ T cells during the immune response to melanoma, we next sought to determine if there was an association between FcγRIIB and other known coinhibitory receptors in melanoma. To test this, we inoculated WT B6 mice with B16-OVA melanoma cells and harvested dLN and spleen for phenotypic analysis 14 days later. CD44^hi^CD8^+^ T cells from the spleen and dLN were analyzed using viSNE (Figure 2A). As expected from our previous results (24), FcγRIIB-expressing CD8^+^ T cells in the spleen (Figure 2B) and dLN (Figure 2C) were contained within regions of high CD44 expression. Interestingly, FcγRIIB was expressed on cells in regions of both high and low CD62L expression, suggesting that FcγRIIB was expressed on both central (CD44^hi^CD62L^hi^) and effector (CD44^hi^CD62L^lo^) memory CD8^+^ T cells. Moreover, viSNE analysis revealed that FcγRIIB^+^ cells were contained within regions of high 2B4 and PD-1 expression in both the spleen (Figure 2D) and the dLN (Figure 2E). To confirm these results, we then used traditional manual gating in FlowJo to assess CD62L, 2B4, and PD-1 expression on FcγRIIB^–^ and FcγRIIB^+^ CD44^hi^CD8^+^ T cells in the dLN and spleen. Consistent with the viSNE analysis, we observed that FcγRIIB^+^ CD44^hi^CD8^+^ T cell populations in the spleen (Figure 2F) and dLN (Figure 2G) contained a significantly reduced frequency of CD62L^+^ cells and a significantly increased frequency of 2B4^+^ and PD-1^+^ cells relative to the FcγRIIB^–^ CD44^hi^CD8^+^ T cell populations.

### Fcgr2b^–/–^ mice have improved antitumor immunity and reduced tumor volume compared with WT mice.

**Figure 3:**
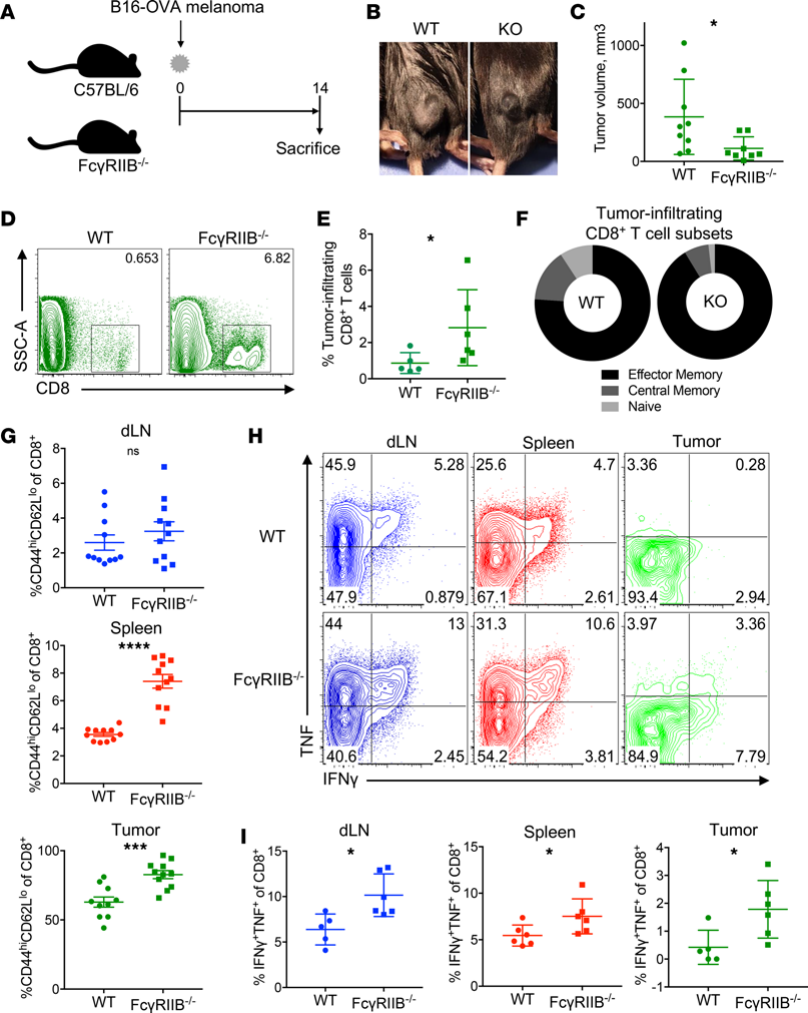
*Fcgr2b*^–/–^ mice have an improved antitumor response compared with WT mice. (**A**) Schematic of experimental design: 10^6^ B16-OVA melanoma cells were subcutaneously injected into the right flank of C57BL/6 (WT) and *Fcgr2b*^–/–^ mice on day 0. Spleen, dLN, and tumor were harvested on day 14. (**B**) Representative pictures of tumor growth in WT and *Fcgr2b*^–/–^ mice. (**C**) Tumor volume in WT and *Fcgr2b*^–/–^mice. (**D**) Representative flow cytometry plots of tumor-infiltrating CD8^+^ T cells in WT and *Fcgr2b*^–/–^ mice. (**E**) Summary data of CD8^+^ T cells within the tumor of WT and *Fcgr2b*^–/–^ mice. (**F**) Differences in CD44^lo^CD62L^hi^ (naive), CD44^hi^CD62L^hi^ (central memory), and CD44^hi^CD62L^lo^ (effector memory) T cell populations in the tumor of WT *Fcgr2b*^–/–^ (KO) mice, represented in mean. (**G**) Summary data of the frequency of CD44^hi^CD62L^lo^ cells of the CD8+ T cell population in the dLN, spleen, and tumor. (**H**) Representative flow plots of CD8^+^ double cytokine producers (IFN-γ^+^TNF^+^) in the dLN, spleen, and tumor of WT and *Fcgr2b*^–/–^ mice. (**I**) Summary data of double cytokine producers (IFN-γ^+^TNF^+^) in the dLN, spleen, and tumor as shown in **H**. Data shown are representative of 2 independent experiments; *n* = 4–6 mice/group/experiment. Mann-Whitney *U* test, *P < 0.05, ***P < 0.001, ****P < 0.0001. dLN, draining lymph node.

Because these data suggested that FcγRIIB expression was associated with a more activated and potentially exhausted phenotype on CD8^+^ T cells, we next queried the functional role of FcγRIIB during antitumor immunity. WT and *Fcgr2b*^–/–^ mice were inoculated with B16-OVA melanoma on day 0. On day 14, tumor volume was measured, and dLN, spleen, and tumor were harvested to assess the CD8^+^ T cell response to the tumor (Figure 3A). Results revealed a significant decrease in tumor volume in *Fcgr2b*^–/–^ animals compared with WT controls (Figure 3, B and C). Consistent with these results, *Fcgr2b* deficiency resulted in a greater frequency of tumor-infiltrating CD8^+^ T cells compared with WT controls (Figure 3, D and E). Moreover, tumors in *Fcgr2b*^–/–^ animals contained a marked increase in the proportion of infiltrating memory (CD44^hi^CD62L^+^ and CD44^hi^CD62L^–^) subsets relative to naive (CD44^lo^CD62L^+^) CD8^+^ T cells, compared with WT animals (Figure 3F). In contrast, we observed no difference in CD8^+^ T cell frequency of memory subset distribution within the spleens of WT versus *Fcgr2b*^–/–^ animals (data not shown). However, spleens (but not dLN) of *Fcgr2b*^–/–^ animals contained a significantly higher frequency of effector memory CD8^+^ T cells (CD44^hi^CD62L^lo^) compared with WT controls (*P* = 0.0079) (Figure 3G). Importantly, the tumors of *Fcgr2b*^–/–^ animals also contained higher frequencies of the effector memory CD8^+^ T cell subset compared with WT controls (Figure 3G).

To determine the functional impact of *Fcgr2b* deficiency on CD8^+^ T cell responses in melanoma, the production of inflammatory cytokines by CD8^+^ T cells was assessed after ex vivo stimulation with PMA/ionomycin. Results demonstrated a significantly increased frequency of IFN-γ^+^ TNF^+^ double cytokine producers within the CD8^+^ T cell populations of the dLN, spleen, and, importantly, within the tumors of *Fcgr2b*^–/–^ animals compared with WT controls (Figure 3, H and I).

### FcγRIIB inhibited tumor-specific CD8^+^ TIL responses in a cell-autonomous manner.

**Figure 4:**
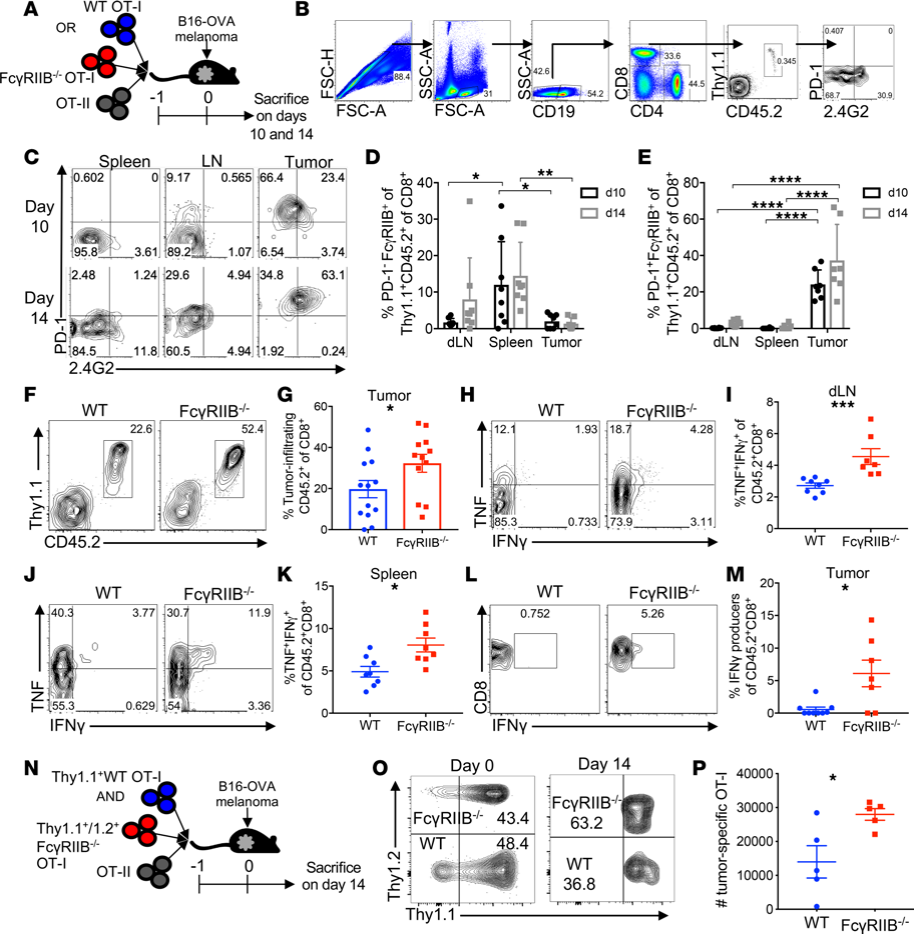
Specific deletion of FcγRIIB on CD8^+^ T cells results in increased tumor infiltration and cytokine production during melanoma. (**A**) Experimental design: 10^6^ WT or *Fcgr2b^–/–^* OT-I and OT-II cells were adoptively transferred into CD45.1^+^ mice. One day later, 10^6^ B16-OVA cells were subcutaneously injected into the right flank. The dLN, spleen, and tumor were harvested on days 10 and 14. (**B**) Gating strategy for analysis of PD-1 and FcγRIIB on OT-I T cells. (**C**) Representative flow plots of PD-1 and FcγRIIB expression on WT OT-I T cells on days 10 and 14 in the spleen, dLN, and tumor. Frequencies of PD-1^–^ (**D**) and PD-1^+^ (**E**) FcγRIIB^+^ OT-I CD8^+^ T cells on days 10 and 14. Representative flow plots (**F**) and summary data (**G**) of tumor-infiltrating WT and *Fcgr2b^–/–^* OT-I cells on day 14. Representative flow plots (**H**) and summary data (**I**) of TNF and IFN-γ^+^ cells among WT and *Fcgr2b^–/–^* OT-I cells in the dLN on day 10. Representative flow plots (**J**) and summary data (**K**) of TNF and IFN-γ^+^ cells among WT and *Fcgr2b^–/–^* OT-I cells in the spleen on day 10. Representative flow plots (**L**) and summary data (**M**) of IFN-γ^+^ cells among WT and *Fcgr2b^–/–^* OT-I cells in the tumor on day 10. Data shown are representative of 2 independent experiments; *n* = 3–5 mice/group/experiment. Two-way ANOVA with multiple comparisons was used in **D** and **E**. Mann-Whitney *U* tests were used in **G**, **I**, **K**, and **M**. (**N**–**P**) 10^6^ MACS-purified (>95% CD8^+^) Thy1.1^+^ WT or Thy1.1^+^/Thy1.2^+^ *Fcgr2b^–/–^* OT-I cells and 10^6^ WT OT-II cells were transferred into C57BL/6 mice. One day later, 10^6^ B16-OVA cells were subcutaneously injected. Spleens were harvested on day 14. Representative flow plots (**O**) of frequencies and (**P**) absolute numbers of Thy1.1^+^ WT vs. FcγRIIB^–/–^ Thy1.1^+^/Thy1.2^+^ CD8^+^ CD44^hi^ T cells. Mann-Whitney *U* test was used in **P**. *P < 0.05, **P < 0.01, ***P < 0.001, ****P < 0.0001. dLN, draining lymph node.

Because FcγRIIB is a known inhibitory receptor on innate immune cells and B cells, the results described above could be due to either a cell-autonomous effect of FcγRIIB inhibitory signals directly on CD8^+^ T cells or an indirect effect of FcγRIIB on APC or B cell populations that impact the CD8^+^ T cell response. Because the cell-autonomous ability of FcγRIIB to regulate tumor-infiltrating CD8^+^ T cell responses has never been tested, we generated TCR-transgenic, antigen-specific OT-I T cells that were deficient in *Fcgr2b*. Thy1.1^+^ WT or *Fcgr2b*^–/–^ OT-I T cells were adoptively transferred into naive CD45.1^+^ congenic recipients 24 hours prior to inoculation with B16-OVA melanoma cells (Figure 4A). Mice were sacrificed 10 and 14 days later and the expression of PD-1 and FcγRIIB was assessed on the WT OT-I T cells in the dLN, spleen, and tumor after a stringent gating strategy (Figure 4B). Results indicated that the spleen had the highest proportion of PD-1^lo^FcγRIIB^+^ OT-I T cells compared with the dLN on day 10 and the tumor on days 10 and 14 (Figure 4, C and D). Due to the high expression of PD-1 on tumor-infiltrating CD8^+^ T cells, we further evaluated the frequency of PD-1^+^FcγRIIB^+^ OT-I T cells in the dLN, spleen, and tumor. We found that the proportion of PD-1^+^FcγRIIB^+^ OT-I T cells was substantially increased in the tumor on days 10 and 14 compared with the spleen and dLN (Figure 4, C and E). These data suggest that although both the spleen and the tumor had higher frequencies of FcγRIIB^+^ CD8^+^ T cells compared with the dLN, the FcγRIIB^+^ CD8^+^ T cells in the spleen were PD-1^lo^, whereas those in the tumor were PD-1^+^. Furthermore, analysis of the tumor-infiltrating lymphocytes on day 14 revealed that *Fcgr2b*^–/–^ CD8^+^ CD45.2^+^ OT-I T cells were present at significantly increased frequencies within the tumor relative to WT OT-I control cells (Figure 4, F and G). Additionally, upon ex vivo restimulation with cognate antigen, we found that tumor-specific *Fcgr2b*^–/–^ CD8^+^ OT-I T cells exhibited a significant increase in the frequency of IFN-γ^+^ TNF^+^ double cytokine producers in both the dLN (Figure 4, H and I) and the spleen (Figure 4, J and K) on day 10. Consistent with many previous studies (25, 26), cytokine production by tumor-infiltrating CD8^+^ T cells was reduced compared with CD8^+^ T cells within the spleen and dLN. TNF secretion was undetectable in both WT and *Fcgr2b*^–/–^ CD8^+^ T cells (data not shown). Importantly, although IFN-γ production was undetectable in WT tumor-infiltrating CD8^+^ T cells (Figure 4, L and M), *Fcgr2b*^–/–^ T cells contained a high frequency (approximately 7%) of IFN-γ–producing effector CD8^+^ T cells on day 10 after tumor inoculation (Figure 4, L and M).

Because the above-mentioned data were obtained after adoptive transfer of WT or *Fcgr2b^–/–^* OT-I T cells into WT hosts, to rule out the effect of any *Fcgr2b*^–/–^ non–T cells on the observed effects, we performed a coadoptive transfer experiment in which purified Thy1.1^+^ WT OT-I T cells were coadoptive transferred at a 1:1 ratio with Thy1.1^+^/Thy1.2^+^ *Fcgr2b^–/–^* OT-I T cells into Thy1.2^+^ B6 hosts (Figure 4, N and O). In this experiment, WT and *Fcgr2b^–/–^* OT-I T cells were responding in the same environment, and thus any differential response could not be attributed to differential environments. As shown in Figure 4P, statistically significantly increased numbers of Thy1.1^+^/Thy1.2^+^ Fcgr2b^–/–^ OT-I T cells were identified in the spleens of coadoptive transfer hosts on day 14 after tumor inoculation compared with Thy1.2^+^ WT OT-I T cells. These results further support the conclusion that FcγRIIB functions in a cell-autonomous manner to inhibit tumor-specific CD8^+^ T cell responses in the context of tumor challenge.

### FcγRIIB was expressed on human CD8^+^ T cells isolated from patients with metastatic melanoma.

**Figure 5:**
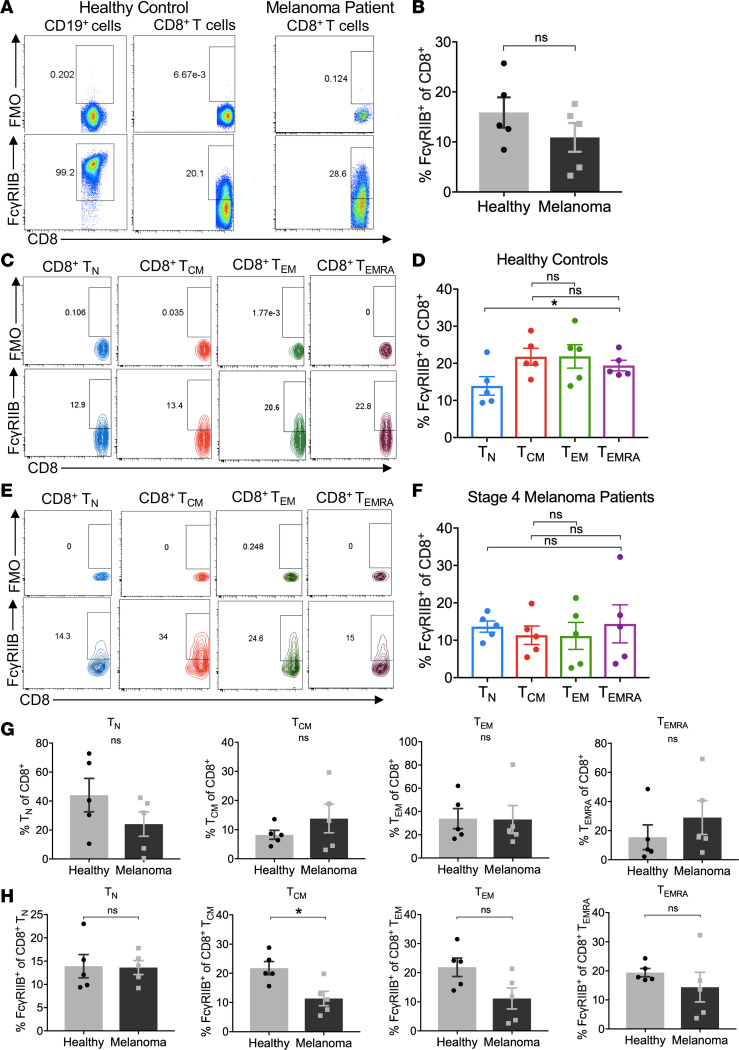
Human CD8^+^ T cells express FcγRIIB. PBMCs were obtained from healthy subjects or patients with stage IV melanoma after informed consent and analyzed directly ex vivo. (**A**) Representative flow plots of FcγRIIB expression (clone FLI8.26) on CD3^+^CD8^+^ T cells compared with FMO and CD19^+^ B cells (positive control). Gating strategy for FcγRIIB comprises gating on singlets, lymphocytes, and live CD3^+^ T cells while excluding CD14^+^, CD19^+^, and dead cells. (**B**) Summary of the frequency of FcγRIIB^+^ CD8^+^ T cells in healthy subjects and patients with melanoma. (**C**) Representative flow plots showing FcγRIIB expression across the following CD3^+^CD8^+^ T cells subsets in healthy donors: naive (Tn), central memory (Tcm), effector memory (Tem), and effector memory expressing CD45RA (Temra). (**D**) Summary of the frequency of FcγRIIB^+^ CD8^+^ T cells across the Tn, Tcm, Tem, and Temra subsets in healthy donors. (**E**) Representative flow plots showing FcγRIIB expression across the Tn, Tcm, Tem, and Temra CD3^+^CD8^+^ T cell subsets in stage 4 treatment-naive patients with melanoma. (**F**) Summary of the frequency of FcγRIIB^+^ cells across the Tn, Tcm, Tem, and Temra CD3^+^CD8^+^ T cell subsets in patients with melanoma. (**G**) Summary of the frequency of the Tn, Tcm, Tem, and Temra CD3^+^CD8^+^ T cells subsets in healthy donors and patients with melanoma. (**H**) Summary of the frequency of FcγRIIB expression of the Tn, Tcm, Tem, and Temra CD3^+^CD8^+^ T cell subsets in healthy donors and patients with melanoma. Data are representative of one experiment with *n* = 5 people per group. One-way ANOVA with multiple comparisons was used to compare more than 2 groups; Mann-Whitney *U* nonparametric, unpaired test was used to compare 2 groups. Error bar denotes mean ± SEM. *P < 0.05. FMO, fluorescence minus one.

Finally, we sought to determine if FcγRIIB was expressed on CD8^+^ T cells in healthy human subjects and patients with melanoma. We acquired whole blood from healthy donors and from treatment-naive patients with metastatic stage III and stage IV melanoma (see Methods for patient characteristics) and isolated PBMCs for phenotypic analysis via flow cytometry. Using an anti-human CD32 mAb and fluorescence-minus-one (FMO) control, we were able to detect FcγRII on human CD8^+^ T cells isolated from both healthy controls and patients with melanoma (Figure 5A). Although this antibody is cross-reactive for both FcγRIIB and FcγRIIA, we recently demonstrated that CD8^+^ T cells isolated from transplant patients contain *FCGR2B* mRNA (24), leading us to conclude that FcγRII staining detected here was likely FcγRIIB expression and not FcγRIIA. The frequency of FcγRIIB-expressing CD8^+^ T cells was not different in the blood of healthy controls compared with patients with melanoma (Figure 5B). We next interrogated the activation status of FcγRIIB-expressing human CD8^+^ T cells. FcγRIIB expression on CD8^+^ T cells isolated from healthy humans was significantly higher on effector memory expressing CD45RA T cells (Temra; CD45RA^+^ CCR7^–^) relative to naive T cells (Tn; CD45RA^+^ CCR7^+^), and trended toward an increase on central memory T cells (Tcm; CD45RA^–^ CCR7^+^) and effector memory T cells (Tem; CD45RA^–^CCR7^–^) relative to naive T cells (Figure 5, C and D). Similar to our mouse data, we found that FcγRIIB was enriched on effector/memory T cell populations compared with naive T cells in healthy human subjects. In patients with melanoma, the frequency of FcγRIIB^+^ cells was similar across CD8^+^ T cell subsets (Figure 5, E and F). In comparing healthy subjects to patients with melanoma, patients with melanoma exhibited similar frequencies of all T cell subsets compared with healthy control subjects (Figure 5G). Of note, frequencies of FcγRIIB^+^ CD8^+^ T cells were decreased within the Tcm subset in patients with melanoma relative to healthy controls and trended toward a decrease in the Tem and Temra subsets in patients with melanoma relative to healthy controls (*P* = 0.06) (Figure 5H). In sum, these data demonstrate that FcγRIIB was expressed and likely played a potentially inhibitory role, on CD8^+^ T cell subsets within healthy human subjects and patients with metastatic melanoma.

## Discussion

Here, we report a cell-intrinsic role of FcγRIIB on tumor-infiltrating CD8^+^ T cells in a mouse model of melanoma. Although the effect of FcγRIIB inhibitory signaling on B cells is well studied, existing dogma has held that FcγRIIB is not expressed on T cells. Here, we report that FcγRIIB was expressed on approximately 30% of CD44^hi^CD8^+^ tumor-infiltrating CD8^+^ T cells. Importantly, using an adoptive transfer approach, we were able to determine that FcγRIIB played a T cell–autonomous role in regulating CD8^+^ T cell accumulation within tumors and regulating CD8^+^ T cell effector function in the context of melanoma. Moreover, this study provides evidence that CD8^+^ T cells in patients with melanoma express FcγRIIB, supporting the notion that this T cell inhibitory pathway could be at play in regulating antitumor responses in humans.

This work is consistent with a previous report demonstrating surface expression of FcγRIIB on CD8^+^ T cells in models of bacterial and viral infection (23). Moreover, 2 recent studies examining the gene expression profile of virus-specific CD8^+^ T cells identified *Fcgr2b* as being a differentially expressed gene (21, 22), showing that the *Fcgr2b* transcript is produced by CD8^+^ T cells (and not solely obtained by trogocytosis of FcγRIIB protein from other cell types). However, whether there was a cell-autonomous functional role of FcγRIIB on tumor-infiltrating CD8^+^ T cells had not been demonstrated. In light of previous data that demonstrated ectopic expression of FcγRIIB on human melanoma cells but found no signaling function of the receptor, it is not a forgone conclusion that FcγRIIB does influence tumor infiltration through CD8^+^ T cell–autonomous inhibitory signaling (27). This study by Sautes-Fridman et al. examined 259 primary and 187 metastatic lesions from 12 different cancer types and noted the selective expression of FcγRIIB by metastatic melanoma cells. They hypothesized that melanoma metastases acquire expression of the inhibitory FcγRIIB as a decoy receptor to serve as a sink for IgG, as a means to escape FcγR-mediated lysis by antitumor IgG antibodies. However, our work demonstrates that on CD8^+^ T cells within the tumor, FcγRIIB had a functional, inhibitory role in suppressing CD8^+^ antitumor immunity.

This work contradicts a long-held dogma that T cells do not express Fc receptors. It is true that the expression level (MFI) of FcγRIIB on CD8^+^ T cells in both mice and humans is substantially lower than that observed in B cells. We speculate that this weak expression may be one reason that FcγRIIB expression on CD8^+^ T cells was not initially appreciated. There are still many unanswered questions regarding the physiologic role for FcγRIIB on CD8^+^ T cells, and we speculate that the differential FcγRIIB expression on B versus T cells may suggest ligation by distinct ligands. Although the best-known ligand for FcγRIIB is the Fc portion of IgG, we recently showed that the immunosuppressive cytokine Fgl2 is an alternate ligand for FcγRIIB on CD8^+^ T cells (24). In addition, other studies have shown that the acute phase proteins C-reactive protein and serum amyloid protein can also bind to FcγRIIB. The relative roles of each of these ligands in signaling through FcγRIIB on tumor-infiltrating CD8^+^ T cells in the context of melanoma remain to be determined. Moreover, although FcγRIIB ligation has been shown to result in apoptosis in B cells, potentially through a SHIP-dependent mechanism (7), the precise mechanisms by which FcγRIIB signaling results in caspase-3/7 activation in T cells is an important outstanding question.

Our data illuminate the role of a checkpoint inhibitor, FcγRIIB, on CD8^+^ T cells in an experimental melanoma model. It is important to note that this tumor model may or may not be representative of other melanoma tumors in mice and/or humans, or of other tumor types, and further studies using additional cancer models are certainly warranted. Ongoing investigation may give rise to innovative strategies in melanoma treatment by targeting the FcγRIIB pathway alone or in conjunction with other coinhibitory pathways. Indeed, because we observed an increase in PD-1 expression on FcγRIIB^+^ cells in the tumor, it is interesting to speculate that ligation of FcγRIIB by anti–PD-1 checkpoint inhibitors (28) could be sending an inhibitory signal to antitumor CD8^+^ T cells, counteracting the efficacy of anti–PD-1 checkpoint blockade. Additionally, similar to the development of bispecific anti-CD19xFcγRIIB antibodies to modulate B cell activation (29, 30), there may be a therapeutic role for bispecific anti-CD8xFcγRIIB antibodies to modulate T cell activation in melanoma treatment. In an era where immunotherapeutic agents now provide durable efficacy and improved outcomes for patients with metastatic melanoma, a thorough understanding of the FcγRIIB pathway and its T cell–autonomous inhibitory role in melanoma-specific immunity will contribute to further improvement in pharmacologic strategies and patient outcomes.

## Methods

### Mice.

B6-Ly5.1/Cr (H2-K^b^, CD45.1) and C57BL/6N (H2-K^b^, CD45.2) mice were obtained from the National Cancer Institute (Charles River, Frederick, Maryland, USA). OT-I (31) and OT-II (32) transgenic mice (C57BL/6 background) purchased from Taconic Farms were bred to C57BL/6 Thy1.1^+^ congenic animals at Emory University. EM:06078 Fcgr2b Fcgr2bB6null B6(Cg)-Fcgr2btm12Sjv/Cnbc (or *Fcgr2b*^–/–^) mice (33) were purchased from the European Mutant Mouse Archive and provided by Academisch Siekenhuis Leiden/Leiden University Medical Center from JS Verbeek (Leiden University Medical Center, Leiden, the Netherlands). B6 embryonic stem cells were used to generate these mice. *Fcgr2b*^–/–^ mice were bred to OT-I transgenic mice at Emory University.

### Adoptive cell transfer.

To monitor antigen-specific CD8^+^ T cell responses, we adoptively transferred OVA-specific transgenic T cells into naive prior to inoculation with B16 melanoma expressing OVA. For the adoptive transfer of tumor-specific CD8^+^ T cells, spleens and mesenteric lymph nodes from CD45.2^+^Thy1.2^+^ *Fcgr2b*^–/–^ OT-I mice or CD45.2^+^Thy1.1^+^ WT OT-I mice were processed into single-cell suspensions. Cells were counted using a Nexcelom Cellometer Auto T4 (Nexcelom Bioscience) and stained with CD8-BV785, CD4-PacBlue, Thy1.1-PerCP, CD45.2-PE/Dazzle, CD45.1-BV605, Vα2-FITC, and Vβ5-PE (all from BioLegend). The frequency of OT-I and OT-II cells was determined via Vα2 and Vβ5 TCR coexpression. Cells were then resuspended in 1× PBS and 1 × 10^6^ OT-I and 1x10^6^ OT-II cells were transferred intravenously into naive congenic CD45.1^+^ B6-Ly5.1/Cr hosts 24 hours prior to tumor inoculation.

### Cancer cell line and inoculation.

B16 melanoma cells engineered to express the OVA epitope (B16-OVA) were provided by Yang-Xin Fu (University of Texas Southwestern, Dallas, Texas, USA) (34). For all experiments, B16-OVA cells were harvested using trypsin, washed in PBS, and counted, and 10^6^ cells were inoculated into the subcutaneous tissue of the right flank. Tumor growth was monitored by daily visual inspection. Tumor volume was calculated using caliper measurement of height, width, and depth.

### Flow cytometric analyses.

Spleen, dLN (right inguinal), and tumor cells from mice were processed to cell suspensions and stained according to manufacturer’s instructions with biotinylated-CD16/32 (clone 2.4G2, BD Biosciences) or a biotinylated isotype control (IgG2bk, BD Biosciences), CD8-BV786 (BioLegend), CD4-PacBlue (BioLegend), CD19-BV510 (BioLegend), CD44-APC/Cy7 (BioLegend), CD62L-PE/Cy7 (BioLegend), Thy1.1-PerCP (BioLegend), CD45.1-BV605 (BioLegend), CD45.2-PE/Dazzle (BioLegend), 2B4-PE (BioLegend), PD-1-PE/Dazzle (BioLegend), and APC Streptavidin (BD Biosciences). FcyRIIB staining on human PBMCs was performed using anti-human CD32 mAb (clone FLI8.26, BD Biosciences). PBMCs were also stained with CD14-BV510 (clone MφP9), CD19-BV510 (clone SJ25C1), CD3-BUV737 (clone UCHT1), CD4-BUV805 (clone SK3), CD8-BUV496 (clone RPA-T8), and CD45RA-Alexa Fluor 700 (clone HI100; all purchased from BD Biosciences) and CCR7-PE/Dazzle (clone G043H7, purchased from BioLegend). For intracellular marker and cytokine staining, cells were incubated for 4 hours at 37°C in the presence of 30 ng/mL PMA, 400 ng/mL Ionomycin, and 10 μg/mL GolgiPlug (BD Biosciences). In some experiments, cells were activated with 30 nM OVA SIINFEKL peptide (GenScript) and 10 μg/mL GolgiPlug (BD Biosciences). After 4 hours, cells were processed using the BD Cytofix/Cytoperm Kit (BD Biosciences) according to manufacturer’s instructions and stained with PE/Cy7 anti-human TNF-α Antibody and Alexa Fluor 700 anti-human IFN-γ Antibody (BioLegend). Absolute cell numbers were determined by CountBright Absolute Counting Beads according to the manufacturer’s instructions (Thermo Fisher). Flow cytometric analysis was performed on an LSRII flow cytometer (BD Biosciences). Data were analyzed using FlowJo 9.9.4 software (Tree Star).

### Human subjects.

Whole blood was acquired from anonymous healthy donors (*n* = 6) and treatment-naive patients with stage IV melanoma (*n* = 6) at Emory University Hospital/Winship Cancer Center. There was no significant difference in ethnicity or sex between healthy subjects and patients with melanoma. Four of six healthy donors identified as Asian and two of six identified as White. Four of five were male (age range 25–63 years). Five of six patients with melanoma identified as White, and one of six identified as African American. Four of six were male (age range 39–78 years).

### Statistics.

All assays were compared using a Mann-Whitney *U* nonparametric test when comparing 2 groups, 1-way ANOVA (with Tukey’s correction for multiple comparisons) when comparing 1 variable across multiple groups, or a 2-way ANOVA (with Sidak’s correction for multiple comparisons) when comparing 2 variables across multiple groups. Statistical analyses were conducted using GraphPad Prism 7. In all figures, data are represented as the mean ± SEM. *P* values of less than 0.05 were considered significant.

### Study approval.

Animal studies were approved by the Institutional Animal Care and Use Committee of Emory University (protocol no. PROTO201700558). This study was carried out in strict accordance with the recommendations in the *Guide for the Care and Use of Laboratory Animals* (National Academies Press, 2011). All animals were maintained in accordance with Emory University Institutional Animal Care and Use Committee guidelines. All animals were housed in specific pathogen–free animal facilities at Emory University. Use of samples from human subjects was approved by Emory University’s Institutional Review Board (protocol no. 00046593) and informed consent was acquired.

## Author contributions

CRF designed and performed experiments, acquired and analyzed the data, and wrote the manuscript. ABM designed and performed experiments, analyzed data, and edited the manuscript. CRF and ABM contributed equally to this work. MT and KBB performed experiments, analyzed data, and edited the manuscript. SP analyzed data. MCL and RK recruited patients with melanoma and edited the manuscript. MLF conceived of the study, obtained funding, designed experiments, analyzed the data, and wrote the manuscript.

## Version 1 02/22/2021

Electronic publication
